# TRP Channels as Molecular Targets to Relieve Cancer Pain

**DOI:** 10.3390/biom12010001

**Published:** 2021-12-21

**Authors:** Milena Duitama, Yurany Moreno, Sandra Paola Santander, Zulma Casas, Jhon Jairo Sutachan, Yolima P. Torres, Sonia L. Albarracín

**Affiliations:** 1 Departamento de Nutrición y Bioquímica, Pontificia Universidad Javeriana, Bogotá 110231, Colombia; sduitama@javeriana.edu.co (M.D.); zcasas@javeriana.edu.co (Z.C.); jsutachan@javeriana.edu.co (J.J.S.); 2 Department of Lymphoma & Myeloma, MD Anderson Cancer Center, The University of Texas, Houston, TX 77030, USA; lymoreno@mdanderson.org; 3 Phytoimmunomodulation Research Group, Juan N. Corpas University Foundation, Bogotá 111111, Colombia; paola.santander@juanncorpas.edu.co

**Keywords:** cancer, pain, TRP channels

## Abstract

Transient receptor potential (TRP) channels are critical receptors in the transduction of nociceptive stimuli. The microenvironment of diverse types of cancer releases substances, including growth factors, neurotransmitters, and inflammatory mediators, which modulate the activity of TRPs through the regulation of intracellular signaling pathways. The modulation of TRP channels is associated with the peripheral sensitization observed in patients with cancer, which results in mild noxious sensory stimuli being perceived as hyperalgesia and allodynia. Secondary metabolites derived from plant extracts can induce the activation, blocking, and desensitization of TRP channels. Thus, these compounds could act as potential therapeutic agents, as their antinociceptive properties could be beneficial in relieving cancer-derived pain. In this review, we will summarize the role of TRPV1 and TRPA1 in pain associated with cancer and discuss molecules that have been reported to modulate these channels, focusing particularly on the mechanisms of channel activation associated with molecules released in the tumor microenvironment.

## 1. Introduction

The tumor microenvironment (TM) can play a crucial role in the production of several signaling molecules that regulate metastasis, tumor growth, and pain. Transient receptor potential (TRP) channels are the central receptors in the transduction of nociceptive stimuli. Molecules from the TM can modulate the activity of TRP channels through the regulation of intracellular signaling pathways. Their modulation is related to the peripheral sensitization observed in patients with cancer, which results in mild noxious sensory stimuli being perceived as hyperalgesia and allodynia.

The expression of TRP channels, such as transient receptor potential vanilloid 1 (TRPV1) and transient receptor potential ankyrin 1 (TRPA1), in cells involved in detecting cancer pain and their role in pain-sensing makes them potential targets for pain relief compounds. In this review, we will describe some possible mechanisms involved in the nociception process associated with cancer that are mediated by factors produced by the TM and which could play an essential role in the direct activation or sensitization of the TRPV1 and TRPA1 channels. In addition, we will discuss the importance of several secondary metabolites derived from plant extracts that could induce the activation, blocking, or desensitization of TRP channels. These compounds could act as potential drug candidates, since their antinociceptive properties may be beneficial in relieving pain derived from cancer.

## 2. The Structure and Physiological Functions of TRP Channels

TRP channels are tetramers formed by subunits with six transmembrane segments (S1–S6), two cytoplasmic domains (NH_2_ and COOH termini), and a loop forming the pore between segments S5–S6 [1]. Most TRP channels are non-selective cation channels [2] that are ubiquitously expressed in mammalian tissues. Differences between TRP channel members are related to the structure of the cytosolic domains, which are characterized by the presence of specific residues in each family [3]. There are seven TRP subfamilies with different biophysical properties: TRPC (canonical), TRPV (vanilloid), TRPA (ankyrin), TRPM (melastatin), TRPP (polycystic), TRPML (mucolipin), and TRPN (no mechanoreceptor potential C channels) [4].

**Figure 1:**
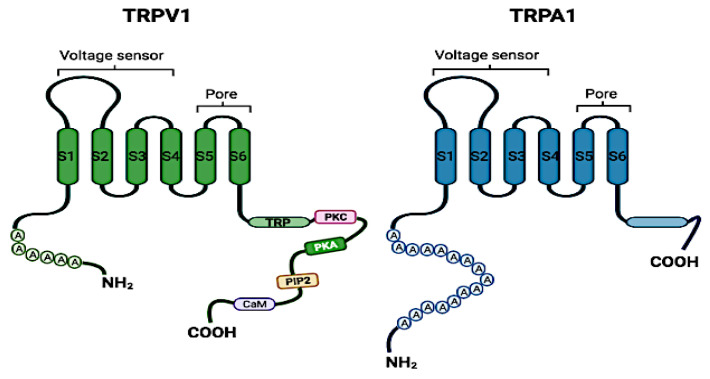
The structure of TRPV1 and TRPA1 channels. The general structure of these two channels includes six intracellular transmembrane domains (S1–S6), as well as carboxy (COOH), and amino (NH_2_) terminals. TRPV1 and TRPA1 share 6 to 16 ankyrin repeats (A) at the amino terminus. Binding sites for protein kinase C (PKC), protein kinase A (PKA), phosphatidylinositol 4,5 bisphosphate (PIP2), and calmodulin (CaM) have been identified on TRPV1 channels. Created with BioRender.com, accessed on 28 June 2021.

TRPV are homo- or hetero-tetrameric calcium channels expressed on the plasma membrane. Based on their homology, the six members of the TRPV subfamily can be classified into four groups: TRPV1/TRPV2, TRPV3, TRPV4, and TRPV5/TRPV6 [4,5,6]. Each subunit usually includes three to five N-terminal ankyrin repeats and a TRP box at the C terminal (Figure 1). TRPV1, TRPV2, TRPV3, and TRPV4 are moderately Ca^2+^ permeable, while TRPV5 and TRPV6 are highly selective Ca^2+^ channels and are strictly regulated by intracellular calcium concentrations ([Ca^2+^]_i_) [7].

Even though TRPV family members have high sequence similarities, they have different gating properties. For instance, TRPV2–6 channels do not respond to temperature stimuli [8], and TRPV2 and TRPV4 are not sensitive to capsaicin [9]. TRPV1 channels are found in pain-sensitive neurons in the dorsal root ganglion (DRG) and trigeminal ganglion (TG) neurons [7], as well as in peripheral small unmyelinated C-fibers, and act as polymodal integrators of noxious stimuli in the skin, muscles, joints, and internal organs [8].

TRPV2 and TRPV4 channels are expressed in DRG neurons, while TRPV3 channels are found in the brain, tongue, testis [6], skin, keratinocytes, and cells surrounding hair follicles [10]. TRPV4 is expressed in non-neuronal cells, such as insulin-secreting β-cells, keratinocytes, smooth muscle cells, and different epithelial and bone cell types [11].

TRPA1, the only member of the TRPA subfamily, is a non-selective cation channel [12,13]. This channel has a calcium-binding domain and a voltage sensor in the C-terminal [14], 16 ankyrin repeat sequences in the N-terminal domain [8,15], and a putative selectivity filter placed at the entrance of the pore (Figure 1). Expression of the TRPA1 channel has been reported in the brain [16], heart [17], small intestines [18], lungs [19], bladder [20], joints [21], and skeletal muscles [22]. Furthermore, high TRPA1 expression has been observed in DRG and TG neurons [23]. In addition, TRPA1 has been reported to be a mechanosensor in peripheral sensory pathways and the inner ear [24,25].

## 3. Activation Mechanisms of TRPV1 and TRPA1

TRP channels can be activated by several ligands, second messengers, reactive oxygen and nitrogen species, and physical stimuli [26,27]. For instance, capsaicin (trans-8-methyl-N-vanillyl-6-nonenamide), endocannabinoids (such as arachidonoyl ethanolamine or anandamide, and 2-arachidonylglycerol (2-AG)), cannabinoids (such as cannabidiol), lipids [28,29], and signaling molecules derived from arachidonic acid (AA) or other similar polyunsaturated fatty acids, can activate TRPs. Other stimuli, such as noxious heat (>43 °C), low pH (<6) [30,31], and membrane depolarization can also modulate TRPs [32,33].

In particular, TRPV1 can be sensitized by inflammatory molecules, such as bradykinin (BK), through G protein-coupled receptors (GPCRs) that generate second messengers such as phosphatidylinositol-4,5-biphosphate (PIP2) [34], inositol triphosphate (IP3), and diacylglycerol (DAG) [35].

Downstream effectors of these second messengers, such as protein kinase A (PKA, also commonly known as cAMP-dependent protein kinase) [36], protein kinase C (PKC) [34,37], Ca^2+^/calmodulin-dependent protein kinase II (CaMKII) [38,39], and AA metabolites, like 12-HPETE [40], subsequently mediate signaling pathways critical for pain regulation. In addition, other pro-inflammatory mediators, such as prostaglandins (PGs) and sympathetic amines, can sensitize TRPV1 nociceptors, thereby, increasing pain sensation or hyperalgesia [41].

TRPA1 contributes to chemo-nociception by acting as a sensor for chemical irritants [42]. Studies have shown that some polyunsaturated fatty acids [43], as well as temperature (17 °C to 40 °C) and pH changes [13,44,45,46] promote the gating of TRPA1 channels [47]. As with TRPV1 channels, different molecules released from inflammatory environments or tissue injury sites have been shown to activate TRPA1 channels [48]. For instance, lipid peroxidation products, and activators of the inflammasome promote the stimulation of TRPA1 channels by an indirect mechanism involving H_2_O_2_ production [49]. Furthermore, endogenous lipidergic metabolites, like epoxyeicosatrienoic acid (EET) and PGs (such as PGA), that are produced in inflammatory environments have also been reported to activate TRPA1 channels [45,50].

## 4. Tumor Microenvironment (TM) and Cancer Pain

The TM modulates cellular growth, metastasis, and angiogenesis through reciprocal communication between cancer cells and its surrounding environment. In this sense, tumor cells can promote interactions between stromal, immune, and vascular cells by releasing growth factors, chemokines, and cytokines [51]. The TM facilitates growth-promoting signals and intermediate metabolites that remodel tissue structure to build the microenvironment.

Other non-cellular components of the extracellular matrix, including collagen, fibronectin, laminin proteoglycans, and various glycoproteins [46,47], are also associated with cancer progression. Thus, the TM is highly complex and dynamic, and interactions between cells occur bidirectionally through cell–cell contacts, as well as through cell-free contacts involving vesicles that transfer information between cells or non-cellular components [52].

Interestingly, these interactions are also related to inflammatory processes. Immune cells infiltrating the tumor, together with fibroblasts and the extracellular matrix, form a scaffold, which supports expansion of the tumor by helping to establish an inflammatory environment, nourish the tumor, and promote its growth [53]. In addition, the hypoxic environment plays a critical role in early tumor development by activating genes associated with the glycolytic pathway in the tumor and inflammatory cells, such as macrophages and granulocytes [54]. Recruitment of these cells into the hypoxic microenvironment also contributes to the hyperproduction of reactive oxygen species (ROS) regulating the NF-κB pathway [55].

**Figure 2:**
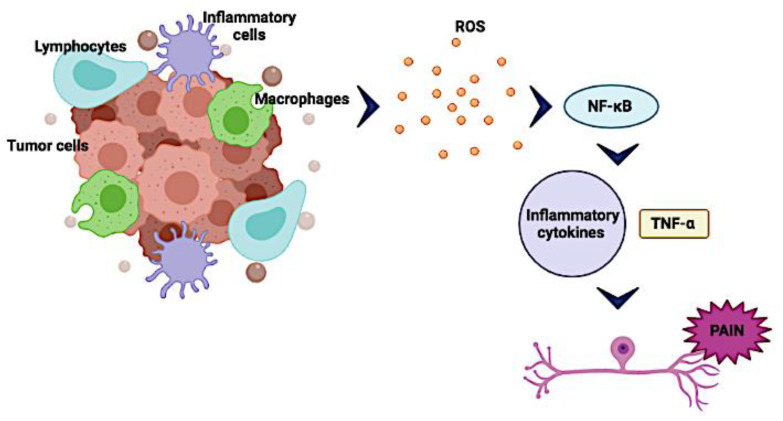
The tumor microenvironment and pain. Interactions between different types of cells present in the tumor are associated with inflammatory processes that induce the release of reactive oxygen species (ROS) regulating the NF-κB pathway within the hypoxic tumor microenvironment. Under these conditions, the activation of NF-κB, particularly in tumor cells and macrophages, induces the secretion of interleukin-6 (IL-6) and tumor necrosis factor-alpha (TNF-α), which sensitize nociceptors associated with pain. Created with BioRender.com, accessed on 28 June 2021.

Under these conditions, the activation of NF-κB in tumor cells [51,56], as well as M1 and M2 macrophages, leads to the secretion of tumor necrosis factor-alpha (TNF-α) and other cytokines, such as interleukin-1 (IL-1), IL-6, IL-12, IL-10, and IL-13 [52,57]. The release of these cytokines initiates and drives the regulated expression of pro-inflammatory mediators and growth factor genes [49], such as chemokines and interferon-gamma [53,54] (Figure 2). In this review, we will discuss in further detail the pro-inflammatory mediators and growth factors that have been associated with the process of nociception.

### 4.1. Pro-Inflammatory Mediators Associated with Nociceptive Processes

As previously mentioned, in malignant tumors, not only cancer cells but also lymphocytes, fibroblasts, and endothelial cells produce and release mediators, such as cytokines, TNF-α, and IL-6 [58], which have been shown to be involved in modulating nociceptors [59]. Activation of these nociceptors has been associated with the onset and persistence of hyperalgesia and allodynia in cancer [60]. For example, an in vitro study using primary cultured TG neurons demonstrated that TNFα upregulated the trafficking of TRPV1 and TRPA1 to the surface of sensory neurons [61].

This study also demonstrated that these channels are transported in neuropeptide-containing, large, dense-core vesicles, which allow them to modulate pain and inflammation [61]. In addition, Fang et al. used a model of bone cancer pain established by the intratibial injection of syngeneic MRMT-1 rat mammary gland carcinoma cells, and found upregulation of IL-6 and its receptors, as well as TRPV1 receptors in DRG neurons. They also reported that the IL-6/JAK/PI3K/TRPV1 signaling cascade may underlie the development of peripheral sensitization and bone cancer-induced pain [62].

Other compounds released in the TM with nociceptive effects include BK and related kinins. Some studies have shown that BK is released in response to tissue injury and plays an essential role in managing acute and chronic inflammatory pain [63]. The action of BK is mediated through the activation of B1 receptors, which are typically expressed at low but detectable levels in sensory neurons, and B2 membrane receptors, which are constitutively expressed at high levels by sensory neurons. Interestingly, B1 receptors are significantly upregulated after peripheral inflammation or tissue injury [64]. Notably, tumor metastasis in the skeleton has been reported to induce significant bone remodeling and tissue damage that leads to the release of BK and indirect sensitization of TRPV1 and TRPA1 channels or direct activation of TRPA1 channels [65].

Several studies have shown that nerve growth factor (NGF) is expressed and secreted by tumor and tumor-associated immune cells and reportedly regulates TRP channels [61,66]. NGF facilitates the innervations of the perivascular nerve to regulate blood circulation in the tumor neovessels supplying its metabolic needs [67]. In addition, NGF has a role in the nociception process as an essential mediator of chronic pain mediated through its interaction with the related receptor tyrosine kinase A (TrkA) [68], although its mechanism of action has not been fully elucidated.

A study by Jimenez-Andrade et al. used a prostate cancer pain model in the marrow space of the mouse femur to induce bone-forming cancerous foci, which allowed the tumor cells to grow and remodel the bone. This model revealed that the tumor-associated stromal cells expressed and released NGF, which drove the pathological reorganization of nearby TrkA+ sensory nerve fibers [69].

Another study with a mouse cancer model simulating the anatomical and functional features found in human oral cancer patients demonstrated that anti-NGF treatment decreased plasma TNF-α and IL-6 levels [70]. These results suggest that NGF can stimulate the release of pro-inflammatory mediators that bind to TrKA and modulate directly or indirectly the activity of TRPV1 and TRPA1, thereby, inducing pain in various malignancies, including breast and prostate cancers [71].

### 4.2. Acidification of the Tumor Microenvironment

Changes in pH have been observed in aggressive cancer cells and are considered to be an additional factor that plays an essential role in the sensitization and activation of nociceptors in the TM [72]. Cancer cells are highly dependent on glycolysis, which releases lactate and H^+^, particularly in deep regions where oxidative phosphorylation is reduced. Metabolic adaptation to hypoxia is driven by the master transcription factor hypoxia-inducible factor (HIF1α), which substantially increases the expression of glucose transporters and various glycolytic enzymes [73].

This metabolic reprogramming provides tumor cells with abundant energy and biosynthetic intermediates when O_2_ availability is limited. HIF1α-mediated upregulation of genes encoding lactate dehydrogenase A (LDHA) and monocarboxylate transporter 4 (MCT4) further supports increased glycolytic flux in hypoxia [74]. Specifically, pyruvate is converted by LDHA to lactate to ensure the regeneration of NAD^+^, while MCT4 favors the passive release of lactate out of cells along its concentration gradient. Importantly, MCT4 is a lactate/H^+^ symporter and plays a critical role in H^+^ output from hypoxic cancer cells [74].

Another essential source of H^+^ ions results from the hydration of CO_2_ through the action of carbonic anhydrase (CA), which generates the majority of H^+^ and HCO_3_^−^ produced in oxidative tumor areas. The production of H^+^, lactate, and HCO_3_^−^ is essential for minimizing the accumulation of cytosolic acid. However, the extracellular accumulation of these species complicates the intracellular elimination of H^+^. Due to CO_2_ being a weaker and more diffusible acid than lactic acid, the contribution of CO_2_ to the reduction of intracellular H^+^ ion concentration, arising from both glycolysis and respiration, can be considered as the most likely mechanism of acid dissipation [75].

Acidification of the TM has also been associated with nociception through the sensitization of primary sensory neurons. DRG neurons are characterized by the expression of pH-sensitive receptors, particularly those that sense acidic pH and contribute to the development of pain associated with cancer. This mechanism may act synergistically with tumor-derived inflammation through the regulation of TRPV1 and TRPA1 channels [70,71].

## 5. The Role of TRP Channels in Cancer Pain

Several molecules including cytokines, such as TNF-α [64,66], IL-1 and IL-6 [76,77], TGF-β and platelet-derived growth factor [67,68], NGF, endothelin [78], PGs, BK, and epidermal growth factor [79], have been reported to directly or indirectly regulate the activity of TRPV1 and TRPA1 channels through the activation of signaling pathways downstream of GPCRs. Furthermore, other pro-inflammatory agents also act through receptor tyrosine kinases (RTKs) by activating phosphatidylinositol 3-kinase (PI3K) and ERK pathways that can induce TRP channel phosphorylation and sensitization by lowering their activation threshold, leading to the development of hyperalgesia and allodynia [80].

The activation of phospholipase C (PLC), PI3K, and adenylate cyclase (AC) have been reported to be involved in the sensitization of TRPV1 and TRPA1 after the binding of BK or NGF receptors [70,71,72,73,74,75]. In this section, we will describe some possible mechanisms that may be involved in the nociception process associated with cancer. These mechanisms are mediated by factors produced by the TM and may have an important role in the direct activation or sensitization of the TRPV1 and TRPA1 channels.

### 5.1. G protein Coupled Receptor (GPCR)-Mediated Modulation of TRPV1 and TRPA1 Channel Activity

Some inflammatory molecules are involved in the sensitization of TRPV1 and TRPA1 through the promotion of PIP2 hydrolysis into the second messengers IP3 and DAG, and subsequent increases in ([Ca^2+^]_i_) [81]. In this sense, diverse GPCRs, such as proteinase-activated receptor 2 (PAR-2) [82], purinergic receptor P2Y1 [83,84], and G-protein coupled 5-HT receptor [85,86], can induce Ca^2+^ release by stimulating the PLC-β pathway [87]. In this way, inflammatory mediators and neurotransmitters that activate PLC-coupled receptors can improve the sensitivity of TRP channels to other physiological stimuli [88].

For instance, it has been reported that activation of PAR-2 by intrathecal application of peptide SLIGKV-NH2 induced thermal hyperalgesia in adult Wistar rats. Interestingly, this response was blocked by pretreatment with the TRPV1 antagonist SB366791 and reduced by treatment with the protein kinase inhibitor staurosporine [89]. Together, these results suggest a functional coupling between the PAR2 and TRPV1 receptors on the central branches of DRG neurons that mediate nociception through TRPV1.

**Figure 3:**
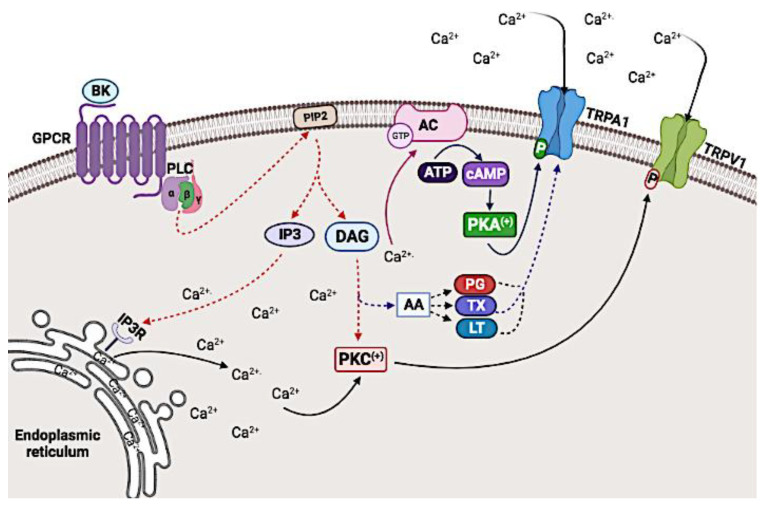
Modulation of the TRPV1 and TRPA1 channels by bradykinin (BK). BK binds to its BR2 receptor, which activates the phospholipase C (PLC) pathway resulting in hydrolysis of phosphatidylinositol bisphosphate (PIP2) into inositol triphosphate (IP3) and diacylglycerol (DAG). IP3 induces the release of Ca^2+^ from the endoplasmic reticulum, thus, increasing the intracellular Ca^2+^ concentration. This increase activates protein kinase C (PKC), which phosphorylates TRPV1 and activates adenylate cyclase (AC) resulting in an increase in cAMP activating protein kinase A that phosphorylates TRPA1. DAG is hydrolyzed into AA, which is the precursor for the synthesis of inflammatory mediators, such as prostaglandins (PG), thromboxanes (TX), and leukotrienes (LC), that activate TRPA1. Created with BioRender.com, accessed on 28 June 2021.

In contrast, binding of BK to its receptor activates PLCβ isoforms resulting in a moderate reduction in PIP2 levels, which releases TRPV1 channels from BK-induced inhibition (Figure 3). In addition, it has been reported that BK-mediated sensitization of TRPV1 is derived from phosphorylation of the residues S502 and S800 by PKC [90,91], suggesting that TRPV1 sensitization is mediated by both events, acting synergistically [92].

However, Lukacs and collaborators reported that maximal stimulation of TRPV1 with capsaicin led to a severe PLCδ-dependent decrease in PIP2 and PIP4 in DRG neurons. The decrease in these lipids parallels an increase in channel desensitization, regulating the TRPV1 channel activity. However, these differences are likely associated with alterations in the bilayer produced by the methodologies used to incorporate PIP2 [93,94].

The role of PIP2 in the regulation of TRPV1 remains unclear. There is strong evidence showing that channel desensitization is associated with both a decrease in PIP2 [95] and an increase in ([Ca^2+^]_i_) [31,79]. Using electrophysiology experiments, Lishko and collaborators showed that the binding of ATP activates TRPV1 and induces a Ca^2+^ influx that activates Ca^2+^-CaM, a channel inhibitor that replaces ATP. Since both share the same binding site, they can induce the inactivation or desensitization of the channel [96]. However, another study using CaM inhibitors and CaM dominant-negative mutants demonstrated no effect on TRPV1 desensitization [97], suggesting that CaM activation is not part of the signaling pathways that regulate this channel.

Another proposed mechanism in TRPV1 desensitization involves the protein phosphatase 2B, also called calcineurin (PP2B) [81,91,98]. PKC phosphorylates and sensitizes TRPV1 through the anchor protein A-kinase 150 (AKAP150) [82,99]. TRPV1 phosphorylation results in sensitization of the channel by multiple pain-inducing stimuli, including noxious heat (>42 °C), acidic pH, and capsaicin. Therefore, channel dephosphorylation is a critical mechanism leading to channel desensitization.

Biochemical and electrophysiological studies have shown that PP2B associates with AKAP150 and TRPV1 in cultured TG neurons and that gene silencing of AKAP150 reduces the baseline TRPV1 phosphorylation. In addition, it has been determined that capsaicin-induced TRPV1 desensitization is sensitive to inhibition of PP2B [100,101] by cyclosporine [100,102]. However, functional studies in neurons isolated from AKAP150^−^/^−^ mice and in heterologous expression systems of TRPV1 showed that the anchor protein is not necessary for the pharmacological desensitization of the channel [103], suggesting the necessity of other molecules.

On the other hand, PGE2 released by tumor cells contributes to nociceptor sensitization by binding to the Gs-protein-coupled receptor subtypes EP3C and EP4 [104], increasing intracellular levels of cAMP and activating PKA [105]. PKA was reported to transiently sensitize capsaicin-activated currents in rat sensory neurons and potentiate heat-activated currents in HEK 293T cells that heterologously express TRPV1 [94] by Ser-116 and Thr-370 phosphorylation.

However, PKA-mediated phosphorylation of TRPV1 also involves a mechanism that decreases Ca^2+^-dependent channel desensitization after capsaicin activation, as was reported by Mohapatra and Nau [106]. A significant decrease in tachyphylaxis was observed when cells were pretreated for 5 min with 10 µM FSK, an AC activator, and therefore indirect activator of PKA. FSK increased the current amplitude after the first application of capsaicin by approximately 47.2–50.5% between the second and fourth applications, respectively.

These findings suggest that PKA-mediated TRPV1 sensitization does not induce channel desensitization, as it does with PKC after the repeated application of capsaicin, indicating that the decreased desensitization observed after TRPV1 phosphorylation could be specific to PKA. This mechanism could contribute to the greater availability of receptors for noxious stimuli and, therefore, to the sensitization of nociceptors [106].

TRPA1 has also been shown to be modulated by bradykinin receptor 2 (BR2), a GPCR [107]. This activation can regulate the activity of TRPA1 via PLC and PKA additively or synergistically [108]. BR2-mediated regulation is thought to occur through two mechanisms. First, PLC activation induces an increase in intracellular Ca^2+^ concentration, which prompts activation of Ca^2+^-sensitive ACs, such as AC1, leading to cAMP [109] and PKA activation.

The second proposed mechanism involves the binding of BK to BR2, activating both Gq- and Gs-signaling pathways. The activation of both PLC mediated by Gq and PKA mediated by Gs/cAMP could contribute to the sensitization of TRPA1 in an additive way [108]. It has been reported that sensitization of TRPV1 by inflammatory mediators rapidly and potently improves channel activation by at least three times via PKA [110] and up to six times via PKC [111].

In contrast, for TRPA1, the PKC-dependent enhancement is substantially lower and short-term or null [108] compared to that of TRPV1 and other TRPs. Similarly, the PKA pathway has a lower effect on the activity of TRPA1 [15]. These observations suggest that PKC activation does not contribute to TRPA1 sensitization, in contrast to the sensitization mechanism proposed for TRPV1.

As mentioned above, the regulation of TRPV1 in nociceptive neurons by protein kinases is dependent on AKAP150. Zimova et al. used HEK 293T cells transfected with cDNA plasmid encoding wild-type or mutant human TRPA1 to show that AKAP150 is also necessary for the effective phosphorylation of TRPA1 by PKA and PKC. These results suggested a basal phosphorylation state or direct effect of AKAP150 on TRPA1 potentiating the channel at negative membrane potentials [112]. On the other hand, analysis of the channel sequence of TRPA1 predicted several phosphorylation sites [99,113], four of which have been considered relevant: S86, S317, and S428 in the N-terminal domain; and S972, located in the C- terminal, close to the transmembrane domain 66 [15].

The proximity of S972 to the channel pore and the TRPA1 voltage sensor [114] may explain how phosphorylation leads to channel sensitization. Phosphorylation on S972 could induce a conformational change that affects the pore by adding a polar phospho-serine to the adjacent sensor, thereby, influencing the activation of TRPA1 by voltage [15]. Thus, phosphorylation of the channel would significantly affect its activation threshold, making nociceptors more sensitive to noxious stimulation by allogeneic and pro-inflammatory agents, such as PGs, histamine, trypsin, and BK. All of these agents are present in the TM and act on GPCRs, stimulating the production of intracellular mediators that directly or indirectly activate TRPA1 [115].

Polyunsaturated fatty acids, such as AA and their metabolites, including PGs, thromboxane (TX), and leukotrienes (LT), activate TRPA1 channels [116,117]. In addition, IP3 induces activation of TRPA1 through an increase in intracellular Ca^2+^ released from the endoplasmic reticulum (Figure 3) [118,119,120,121,122]. Currently, the mechanisms that mediate cancer-induced nociception are not well-defined. However, studies have shown that inflammatory pain models provide a promising approach to study the factors involved in the development of pain in cancer. Activating these pathways sensitizes or desensitizes these channels through different controversial mechanisms and allows further understanding of this type of pain, as well as the development of possible therapeutic alternatives.

### 5.2. Modulation of the Activity of the TRPV1 and TRPA1 Channels Mediated through Receptor Tyrosine Kinase A (TrkA)

NGF has been reported to cause hyperalgesia that can last up to days through interactions of its high-affinity receptor TrkA with TRPV1. Downstream, TrkA activates the MAP kinase (MAPK), PI3K, and PLCγ pathways. However, it is not clear whether all three pathways are directly involved in NGF-induced pain [103,104,105,107,108,109].

The activation of PLCγ, mediated through the binding of NGF to TrkA, was initially reported to play a critical role in channel sensitization to heat responses in adult rat DRG neurons [123]. However, other studies in DRG neurons have suggested that NGF-dependent sensitization could involve the PI3K pathway, and not the ERK or PLCγ pathways [124]. An additional study using adult rat DRG neurons demonstrated that both PI3K and ERK activation mediate inflammatory heat hyperalgesia through TRPV1 sensitization in the presence of extracellular Ca^2+^ [125].

Likewise, studies by Zhu and Oxford support a role for the PI3K and MAPK pathways, and not the PLCγ pathway, in the acute sensitization of TRPV1 by NGF in response to repeated application of capsaicin. This response was detected in the presence or absence of extracellular Ca^2+^. An increase in Ca^2+^ input currents of 2.09 times and 4.23 times was observed in DRG neurons and CHO cells expressing heterologous hTrkA-TRPV1, respectively, as well as downstream PKC activation, which contributed to channel sensitization [124].

These studies also found that pre-treatment with PD98059 and LY294002, inhibitors of the ERK and PI3K pathways, respectively, completely blocked the sensitization effect to NGF, and improved the desensitization of TRPV1, which occurred after the repeated application of capsaicin [107,111,112].

Although PI3K and ERK have generally been studied as independent pathways that fulfill different physiological functions [114,115], growing evidence suggests that both pathways may be related [114,116,117,126]. PI3K inhibition has been reported to block capsaicin- and NGF-induced ERK activation in adult rat DRG neurons. This inhibition suggests that PI3K acts in an ERK-dependent manner, as observed in acute TRPV1 sensitization by NGF [124] and capsaicin-induced heat hyperalgesia, where PI3K was involved in the early events of hyperalgesia and ERK in its long-term maintenance [125].

Recently, Stein and colleagues reported that NGF acts through the PI3K pathway but not through the PLC pathway to facilitate the traffic of TRPV1 to the plasma membrane and its function during hyperalgesia. The authors reported that traffic regulation is mediated through the physical and functional interaction of PI3K-p85β with the channel. They also found that enzymatic conversion of PIP2 to PIP3 by the PI3K pathway promotes channel activation, and that although PIP2 is not an inhibitor of TRPV1 channels, it does potentiate activity in both heterologous cells and DRG neurons [91]. This last study increases the controversy surrounding the role of PIP2 in channel modulation, since the data contradict the PLC model of NGF-mediated sensitization of TRPV1, which suggests that PIP2 inhibits TRPV1, while hydrolysis of PIP2 by PLC alleviates this inhibitory effect [127].

Interestingly, this is not the first study to contradict this model of TRPV1 regulation. Previously, the perfusion of PIP2 in excised plasma membrane patches expressing TRPV1 channels has been reported to increase the effect of capsaicin, indicating that PIP2 has a positive regulatory role. The controversy regarding the role of PIP2 activity is likely associated with the methodologies used to incorporate PIP2 or liposomes versus perfusion [93,94]. Thus, although the mechanism involved in the hydrolysis of PIP2 by PLC and its role in the TRPV1 sensitization process remains unclear, the evidence thus far indicates that both the PI3K and ERK pathways are involved in NGF-mediated TRPV1 sensitization in adult rat DRG neurons.

NGF-mediated activation of TrkA may also be involved in regulating TRPA1 during p38 MAPK-dependent cold hyperalgesia in DRG neurons. In these neurons, an increase in channel expression was observed after inflammation, which coincided with the appearance of greater sensitivity to cold (5 °C). These findings suggested that inflammation increases TRPA1 expression in DRG neurons through activation of TrkA receptors and p38 kinase, and contributes to hyperalgesia associated with damaging cold [128].

**Figure 4:**
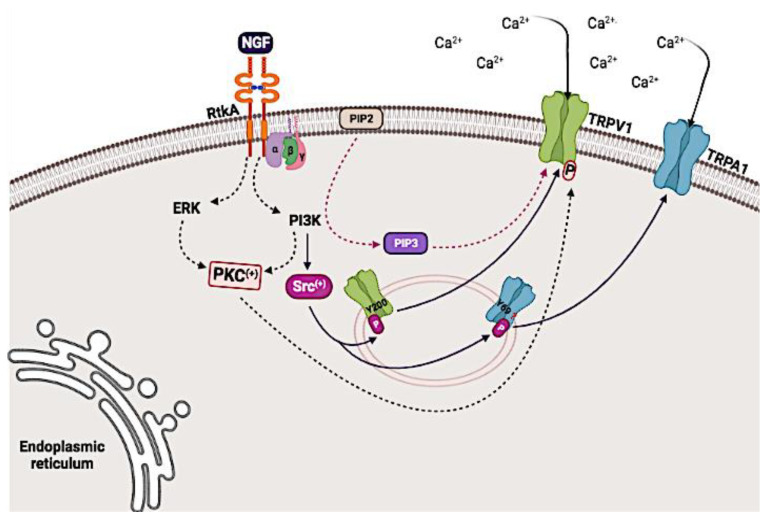
TRPV1 and TRPA1 channel sensitization mechanisms induced by nerve growth factor (NGF). NGF binds to its TrkA receptor to activate the MAPK-ERK and PI3K pathways, which activate protein kinase C (PKC) resulting in the phosphorylation and sensitization of the TRPV1 channel. Likewise, activation of PI3K mediates the enzymatic conversion of phosphatidylinositol bisphosphate (PIP2) to phosphatidylinositol triphosphate (PIP3), which promotes activation of TRPV1. Activation of PI3K leads to the activation of Src, which facilitates the trafficking of TRPA1 and TRPV1 towards the membrane. Created with BioRender.com, accessed on 28 June 2021.

Likewise, the binding of NGF to TrkA activates the PI3K pathway. One element of this signaling cascade is the tyrosine-protein kinase Src, which phosphorylates both TRPV1 and TRPA1 [70,129,130]. The residues Y69 [131], Y22, and Y680 in the N-terminal cytoplasmic domain of TRPA1 have been shown to be phosphorylated by Src (Figure 4). In addition, Src was reported to phosphorylate residue Y200 on TRPA1, leading to an increased channel insertion into the plasma membrane [132]. For this reason, this pathway has also been related to the upregulation of TRPA1 mediated by NGF [70,129].

A study by Diogenes and collaborators used an oral-facial pain model in adult male rats and demonstrated that a subcutaneous injection of 1 mg/kg of NGF upregulated the expression of TRPA1 in the nociceptors of the TGs sensitive to capsaicin [93]. Similar results were also reported in an oral cancer pain model, where NGF was found to upregulate the expression of both TRPA1 and TRPV1 [70]. It is believed that this positive regulation induced by NGF is closely associated with post-transcriptional modifications that regulate expression of the channels and play an essential role in the maintenance of pain associated with cancer.

In summary, these results suggest that NGF acts through the ERK and PI3K pathways to sensitize TRPV1 and TRPA1 channels, and induce hyperalgesia by heat or cold. Activation of PI3K stimulates trafficking of both channels towards the membrane, which increases their membrane expression as well as their ability to be activated or sensitized by NGF and other molecules released by the tumor. Translocation of the channel to the membrane induces the increased activity of DRG neurons that express these channels resulting in a persistent pain sensation in cancer patients.

### 5.3. Other Mechanisms

In addition to activating signaling pathways, stimuli, such as pressure and pH changes, which modulate TRP channel activity, are involved in the development and persistence of pain in cancer. Tumor expansion causes pressure on surrounding organs, generating visceral pain. Several studies have reported the role of TRPA1 in mechanical sensory stimulation, particularly in harmful mechanotransduction due to nerve damage, cancer treatment, and inflammation [133]. Lennertz and collaborators reported that a subset of cutaneous primary afferent C-fibers exhibited sensitization to mechanical stimuli in inflamed tissue.

This mechanical sensitization depended on TRPA1 channel activation, since inhibition of this channel in nerve fibers that express TRPA1 significantly alleviated this hypersensitivity [134]. Based on these results, it has been suggested that primary sensory neurons contribute to inflammatory mechanical hyperalgesia through a mechanism involving TRPA1. Recently, Moparthi and Zygmunt found that human irritant receptor TRPA1 (hTRPA1) channels reconstituted in artificial lipid bilayers are sensitive to changes in bilayer pressure.

Interestingly, this effect was maintained in channels with and without N-terminal ankyrin repeats (ARD), suggesting that ankyrin repeats are unnecessary for the TRPA1 response to mechanical stimuli [127,135]. However, these findings do not exclude the role of N-ARD in the coordination of mechanical stimuli and channel activity in a native environment via interactions with cell membrane lipids. Furthermore, any interaction between the channel’s N-ARD cysteines and electrophilic compounds or ROS, such as H_2_O_2_, present in the TM [125,136], could change the conformation of the protein, possibly affecting TRPA1’s mechanosensitivity [133].

As previously mentioned, the low pH observed in aggressive cancer cells is an additional factor that plays a critical role in the sensitization and activation of nociceptors in the tumor environment. An increase in glucose consumption and lactate production decreases the pH at the intracellular level. Tumors regulate pH through MCT1 and MCT4, Na^+^/H^+^ exchangers, anion exchangers, carbonic anhydrases, V-H^+^-ATPase, Na^+^/HCO_3_^−^ cotransporters, and HCO_3_^−^ transporters, creating an acidic extracellular cancer environment [137] with values as low as pH 6.5 [123,138].

Of note, cancer cells that originate in several different tissues, such as the prostate, breast, and lung, have a propensity to metastasize to the bone microenvironment [139]. The tumor burden within the bone causes unbearable breakthrough pain with properties of continuous pain [140]. Bone colonized by solid cancer generates an acidic extracellular microenvironment mediated by osteoclasts releasing protons and hydrochloric acid to degrade bone minerals during bone resorption [141].

The acidosis is allogenic for sensory neurons. TRPV1 and TRPA1 could be involved in the development of cancer-associated pain, mediated by the acidic tumor environment. For example, extracellular acidification has been reported to activate TRPV1 with a pH below 6.4 and sensitize the channels at pHs greater than 7.4 [31]. This induces a shift in its activation threshold towards lower temperature or ligand concentration, through the protonation of the extracellular Glu residue (E600) located between the fifth transmembrane domain and the putative pore-forming region [142].

In addition to the modulation by extracellular proton concentration, TRPV1 activity is also regulated by intracellular acidification in both heterologous expression systems and nociceptive DRG neurons. In conditions where the external pH is neutral, the intracellular acidification induced after channel activation by capsaicin was strictly coupled to a Ca^2+^ input component [143], which could be mediated through any of the following three proposed mechanisms. First, a mitochondrial Ca^2+^ clearance pathway that leads to negative potential collapse across the inner mitochondrial membrane and subsequent TRPV1 proton leakage [144]; second, the proton displacement of Ca^2+^ buffer proteins [91,128]; and third, an antiport of Ca^2+^-H^+^ by Ca^2+^-ATPases, in the semipermeable membrane that encloses the cytoplasm [145].

Although it remains unclear as to which of these mechanisms mediates intracellular acidification after the activation of TRPV1, the evidence shows that this effect could also activate TRPA1 channels. Wang and collaborators found that intracellular acidification by weak acids, such as carbonic, acetic, and propionic acid released protons into the intracellular medium, thereby, activating TRPA1 in sensory TG neurons [146]. These results suggest that, in a tumor environment, acidification of the extracellular medium could induce a synergistic effect between TRPV1 and TRPA1.

After activation of TRPV1, the decrease in intracellular pH would be sensed by TRPA1, and induce their activation. These effects result in the potentiation of the nociceptive sensory neurons that co-express both channels. Recently, de la Roche and collaborators reported that extracellular acidosis activated and sensitized the hTRPA1 through a mechanism that did not involve modification of N-cysteine intracellular terminals as essential sites of interaction for TRPA1 electrophilic agonists [45]. This finding further implicates TRPA1 in proton-induced pain transduction, particularly in cancer-associated pain.

## 6. The Role of TRP Channel Agonists and Antagonists in Cancer Pain

TRPV1 and TRPA1 are found in nociceptors that transduce information associated with noxious stimuli and participate in pain response. Therefore, regulating TRPV1 and TRPA1 channel activity with antagonists or stimulating them with agonists until they become desensitized could result in a strategic tool that would promote an analgesic effect [147].

TRPA1 antagonists have been used in different pain models and have shown a higher analgesic activity and common side effects. Studies in diabetic mice, for example, have shown that some TRPA1 antagonists improve mechanical hyperalgesia and prevent the loss of skin nerve fibers [43,139]. Such findings have led to the identification and evaluation of new molecules in preclinical and clinical studies. Two synthetic antagonists, CB-625 and GRC15736, have been tested in models of acute post-surgical pain and diabetic peripheral neuropathy with promising results [147].

Since TRPA1 channels have been shown to be important in the transduction of pain in cancer, evaluations of these antagonists in pain cancer models would be valuable. However, the use of some synthetic antagonists, particularly in TRPV1 channels, has been reported to increase the heat pain threshold after their administration, generating scalding lesions [134,148,149]. In other cases, the development of hyperthermia, which lasted up to 4 days, with body temperatures close to 40.2 °C was reported [147]. Hyperthermia has been shown to occur through TRPV1 activation due to an increase in the concentration of hydronium ions [150].

Therefore, antagonists that interfere with acid pH-mediated activation of TRPV1 could be used to inhibit the effect of hydronium ions. However, this concept is still controversial, as it is true for some compounds, such as AS1928370 and A-1165442 [137,150], but not for others including PHE377 and JTS-653 [150,151,152]. Thus, further studies are required to elucidate the mechanism of action of these compounds on channel activity, particularly in a tumor environment, which, as previously described, is characterized by a decrease in extracellular pH.

In contrast to the challenges associated with the development of antagonists, agonists have been used clinically to locally desensitize TRP channels with only mild side effects, such as irritation. This notion of a paradoxical use of TRP channel agonists as pain regulators has been derived from the experience of specific herbal remedies used in traditional medicine. For example, preparations or recipes containing TRP channel agonists, such as cinnamaldehyde (TRPA1) or shogaol (TRPV1), have long been used topically or orally to relieve neuralgia, arthralgia, menstrual pain, and headaches [147].

As mentioned above, capsaicin, the main pungent component in the fruit of the plants of the genus *Capsicum*, is the primary agonist of TRPV1 [153]. Capsaicin is a naturally occurring alkaloid member of vanilloid compounds, such as vanilla, vanillin, eugenol, and zingerone [154]. Capsaicin initially activates TRPV1, but after repeated stimulation, it causes a long-lasting refractory period, termed desensitization [155].

This process has been studied for its therapeutic potential in managing pain, such as osteoarthritis and postsurgical pain [153,156]. An analgesic effect has been reported possibly attributed to the degeneration of epidermal nerve fibers [157], which could be mediated by apoptosis through the activation of caspases. However, the mechanisms by which the application of capsaicin induces this degeneration in sensory fibers have not been established [158].

Currently, topical formulations of capsaicin are widely used for the treatment of pain. It has been reported that low doses of capsaicin between 0.025% and 0.075% are not particularly effective in treating different forms of chronic neuropathic pain and musculoskeletal pain. However, it does seem to have a clinical benefit for pain treatment [159] in postherpetic neuralgia [159,160], and painful diabetic neuropathy [161]. Furthermore, the use of topical capsaicin at 0.075% applied four times a day for three weeks caused degeneration of skin nerve fibers and, consequently, decreased sensitivity to cold and tactile stimuli, but not to heat and mechanical stimuli [157].

In contrast, the use of topical capsaicin at doses between 5% and 10%, together with the application of local anesthesia, were shown to provide significant relief from neuropathic pain, post-traumatic neuropathy, postsurgical neuralgia, and mixed pain syndrome [161,162,163]. In addition, 8% capsaicin topical patches are available under Qutenza or Transacin for use in patients with postherpetic neuralgia and peripheral neuropathy [164]. However, diverse adverse effects have been reported, including pain, pruritus, skin erythema, papules, and a transient increase in blood pressure [165].

Capsaicin has been used by patients with cancer pain derived from surgeries, chemotherapy, and radiotherapy. The topical use of capsaicin 0.075%, four times a day, has been reported in patients with neuropathic pain resulting from surgery, with good acceptability of the treatment despite side effects [166]. Likewise, temporary pain relief has been reported after using oral capsaicin in patients with mucositis due to chemotherapy or radiotherapy [167].

Finally, the use of 8% capsaicin patches was reported in patients with post-traumatic neuropathic pain with an 80% reduction in allodynia up to 18 months after its application [162]. Although capsaicin induces some adverse side effects, it is well-tolerated, and thus pharmacological strategies, such as the use of anesthetics that attenuate or improve discomfort, coupled to the application of capsaicin have been implemented.

Resiniferatoxin (RTX) is another TRPV1 agonist that has shown promising effects for pain management, particularly in bone cancer. This ultra-potent capsaicin analog, present in the latex of a cactus-like plant, *Euphorbia resinífera*, mimics most of the biological characteristics of capsaicin, with a potency of approximately 1000 times, which induces channel desensitization [168].

In animal models, RTX treatment was found to induce the desensitization of TRPV1-expressing neurons and improved bone cancer pain, providing relief for several weeks in mice and dogs with advanced osteosarcoma [153,154,155,156]. This effect was mimicked by the pharmacological blockade of the TRPV1 gene and channel by the selective antagonist JNJ17203212 [169]. In addition, epidural application of RTX is well tolerated at doses that induce analgesia (0.265 mg in rats), although some adverse effects have been reported (sedation or hyperventilation) at doses approximately 10-times higher, as well as death at doses greater than 10 mg [170].

Clinical studies are currently underway that involve the administration of 3 to 26 µg of intrathecal RTX [171] in patients over 18 years of age with advanced stages of cancer and chronic and intractable pain (clinicaltrials.gov, NCT00804154). Patients who received the lowest doses of RTX experienced varying levels of pain relief, while patients who received RTX injections of 13 or 26 μg reported less pain and better mobility [171].

Furthermore, the reduction in thermal perception was consistent with the death of TRPV1-expressing neurons, without any other sensory or motor changes after treatment. These findings suggest that intrathecal RTX administration can selectively induce cell death in pain-transmitting neurons [172], which is consistent with the reported ability of RTX to alleviate pain [160,173]. These results have not only provided the basis for the development of new specific analgesics for managing this type of pain, but have also contributed to the expansion of novel approaches to identify compounds of plant origin, particularly species that have traditionally been used for pain management.

Many secondary metabolites are present in plant extracts and can directly modulate the activity of the TRPV1 and TRPA1 channels (Table 1). Some traditional plants used for the treatment of pain also have one or more of these metabolites. Compounds, such as tannins, anacardial acid present in extracts of *Anacardium occidentale L* [174], eugenol and linalol identified in extracts of *Bidens Pilosa* [175,176], camphor and linalool from *Piper aduncum* extracts [177,178], and carvone and geraniol from *Lippia alba* [179], may be involved in the potential analgesic effects of these extracts [174,180,181]. However, further studies are required to elucidate the mechanisms mediating the observed effects [181].

These findings allow the identification of new compounds, which may have the potential to regulate these channels and could, therefore, become pharmacological candidates, for example, to treat cancer pain, either as new analgesics or as adjuvants to cancer treatment. A promising alternative in this field would be the use of plant extracts traditionally used to manage different types of pain, which consist of compounds with antinociceptive effects [196].

## 7. Conclusions

The mechanisms associated with cancer pain have not yet been fully elucidated, due to the various molecules and complex processes, which may be involved in its generation and progression. However, we propose that mechanisms that participate in the nociception process in pain are modulated by TRPV1 and TRPA1 channels. Evidence suggests that inflammatory mediators, as well as the enhanced acidification of the extracellular and intracellular components of the TM, regulate the activity of these channels through signaling pathways.

The PLC, PI3K, and MAPK-ERK signaling pathways, activate downstream kinases, such as PKC and PKA, which phosphorylate TRPV1 and TRPA1 channels and promote channel sensitization and reduction of their activation threshold, potentially contributing to the pain sensation experienced by cancer patients. Further evidence suggests that secondary metabolites from traditional plants used for pain management can regulate the activity of these channels. Thus, it would be interesting in the future to examine new molecules with potential antinociceptive properties that are associated with TRPV1 and TRPA1 modulation. The identification of such molecules could lead to the development of treatment strategies with enhanced pain-relieving effects and fewer side effects than the analgesics currently available.

## Figures and Tables

**Table 1:** Plant-derived compounds that could potentially modulate the activity of the transient receptor potential channels, TRPV1 and TRPA1.

Channel	Compound	Source	Activity		Reference
			Agonist	Antagonist	
TRPV1	Capsaicin	*Capsicum*	†		[30]
	Piperine	*Piper nigrum*	†		[182]
	Resiniferatoxin	*Euphorbia resinifera*	†		[30]
	Evodiamine	*Evodia rutaecarpa*	†		[183]
	Cannabidiol	*Cannabis sativa*	†		[184]
	Polygodial	*Polygonum hydropiper*	†		[185]
	Isovelleral	*Lactarius vellereus*	†		[186]
	Tapsigargina	*Thapsia garganica*		†	[187]
	Yohimbine	*Pausinystalia yohimbe*		†	[188]
TRPA1	Allyl isothiocyanate	Wasabi, mustard oil	†		[189]
	Benzyl isothiocyanate	Yellow mustard	†		[189]
	Phenylethyl isothiocyanate	Brussels sprouts	†		[189]
	Isopropyl isothiocyanate	Nasturtium seeds	†		[189]
	Methyl isothiocyanate	*Capparis spinosa*	†		[189]
	Cinnamaldehyde	*Cinnamomum cassia*/*Cinnamomum zeylanicum*	†		[126]
	Methyl salicylate	Wintergreen oil	†		[126]
	Carvacrol	*Origanum genus*	†		[190]
	Tetrahydrocannabinol	*Cannabis genus*	†		[189]
	Menthol	*Mentha*		†	[191]
Both of them	Eugenol	Clove oil	TRPV1/TRPA1		[192]
	Gingerol	*Zingiber officinale*	TRPV1/TRPA1		[126,193]
	Camphor	*Cinnamomum camphora*	TRPV1	TRPA1	[194]
	Allicin	*Allium sativum*	TRPV1/TRPA1		[195]

† Compounds that modulate TRPV1 and TRPA1 as agonists or antagonists.
